# Duty of the Doctor to the Dying

**Published:** 1878-03-20

**Authors:** E. L. Smith

**Affiliations:** Texas


### DUTY OF THE DOCTOR TO THE DYING.

By E. L. Smith, M.D., of Texas.

In reply to the questions propounded by the Virginia practitioner in your December number relative to the duties of the Doctor to the dying, I have a few succinct and explicit ideas to promulgate. The Doctor, after picturing the heart-rending scenes of his case, asks: "Would it be proper to administer one or two inspirations of chloroform under the circumstances?" I occupy the position that there would have been no immorality in so doing. Now, while I do not profess to be a fatalist, or an over-daring and venturesome practitioner, I make no hesitancy in asserting that the chloroform in the case presented by our medical brother, should have been administered; not with the view of extinguishing life or closing the horrible scene; but with the view of mitigating the excruciating tortures of the hopeless sufferer. Nor is it supposable that in such a case the chloroform, unless carried to extremes, would destroy life, but would only have a palliative influence, enabling the poor sufferer to depart unconscious of mental or physical anguish.

It is well understood that we do not profess to cure the thousand and one ills to which humanity is subject, and that where a cure is impossible it is our province to palliate. By administering chloroform to such a patient as the one spoken of, the practitioner would be guilty of no moral terpitude, and need suffer no remorse, but feel rather a sense of comfort in the reflection that he had bestowed the balm of sweet unconsciousness to a dying fellow mortal!

